# Supplementary material for: Differences in medical costs for end-of-life patients receiving traditional care and those receiving hospice care: A retrospective study
Source: PLoS One. 2020 Feb 20;15(2):e0229176. doi: 10.1371/journal.pone.0229176 (PMC7032706; doi:10.1371/journal.pone.0229176)
Supplement: Attachment — Submitted filename: Rebuttal letter.docx [file pone.0229176.s002.docx]

**“Differences in Medical Costs for End-of-Life Patients Receiving Traditional Care and Those Receiving Hospice Care: A Retrospective Study” (PONE-D-19-21828) by Huang et al., for publication in PLOS ONE**

**Journal Requirements:**

#1. When submitting your revision, we need you to address these additional requirements. Please ensure that your manuscript meets PLOS ONE's style requirements, including those for file naming.

Remedy: Thanks for your reminder. We have made sure that the revised manuscript meets PLOS ONE’s style requirement.

#2. We note that you have included the phrase “data not shown” in your manuscript. Unfortunately, this does not meet our data sharing requirements. PLOS does not permit references to inaccessible data. We require that authors provide all relevant data within the paper, Supporting Information files, or in an acceptable, public repository. Please add a citation to support this phrase or upload the data that corresponds with these findings to a stable repository (such as Figshare or Dryad) and provide and URLs, DOIs, or accession numbers that may be used to access these data. Or, if the data are not a core part of the research being presented in your study, we ask that you remove the phrase that refers to these data.

**Remedy:** We have made all the relevant data available in the manuscript and removed the phrase “data not shown,” Thanks for your advice. (Please refer to “Results,” the 1^st^ paragraph, pp.13.)

#3. In your Data Availability statement, you have not specified where the minimal data set underlying the results described in your manuscript can be found. PLOS defines a study's minimal data set as the underlying data used to reach the conclusions drawn in the manuscript and any additional data required to replicate the reported study findings in their entirety. All PLOS journals require that the minimal data set be made fully available. For more information about our data policy, please see http://journals.plos.org/plosone/s/data-availability.

**Remedy:** We will update your Data Availability statement to reflect the information you provide in your cover letter.

#4. In the ethics statement in the manuscript and in the online submission form, please provide additional information about the patient records used in your retrospective study. Specifically, please ensure that you have discussed whether all data were fully anonymized before you accessed them and/or whether the IRB or ethics committee waived the requirement for informed consent. If patients provided informed written consent to have data from their medical records used in research, please include this information.

**Remedy:** This study was reviewed and approved by the Institutional Review Board of Camillian Saint Mary’s Hospital Luodong (approval # SMHIRB104004). The study design conformed to the ethical guidelines of the 1975 Declaration of Helsinki. The written informed consent was waived because the database was provided and analyzed anonymously.

We have added this information to the text. Thanks for your advice.

(Please refer to “Materials and Methods-, Data Collection and Ethical Considerations,” the 2^nd^ paragraph” pp.10).

**Response to Reviewers**

Thank you for the generally positive comments on our manuscript. We also completely agree with your excellent comments. Other responses to your specific comments are listed below.

**Response to Reviewer 1**

### #1. I think the analysis nicely provides a reference on cost for stakeholders. I am not sure, however, that “cost-effectiveness” is what is being measured. Cost-effectiveness examines the costs and outcomes of one or more interventions. So here, I think the intervention is hospice care, and the health outcome is death. From the analysis as written, assessing cost-effectiveness would mean assuming that the outcome of death (matched so carefully between cases and controls) was inevitable. I am not sure that is the case – different therapies could have different effects on patients and their length of survival.

### Remedy: Thanks for your advice. We agree with your opinion. The current study mainly aimed to explore the cost-saving after receiving hospice care rather than cost-effectiveness analysis. In the current study, the intervention is hospice care, and the health outcome is death. If death is inevitable, the current study could not examine different therapies that could have different effects on patients and their length of survival. To avoid making you misunderstand, I substituted the cost-saving from cost-effectiveness. (Please see the “Introduction,” the 4^th^ paragraph” pp.7).

#2. From the data source from the National Health Insurance database, is there information available on the causes of death? I saw that the cases were matched using cancer diagnosis, but did not see data on cause of death. If, for instance, one group had different causes of death than the other (i.e., related to but not definitively their specific cancer diagnosis), that could be an interesting finding. It could also have effects on cost-effectiveness.
**Remedy:** Thanks for your advice. Due to the unavailability of cause-of-death information in the health insurance database, the acquirement of the information for the cause of death was our limitation. Future, the relation of the cause-of-death and medical expenses in EOL was suggested. (Please refer to “Discussion,” the 8^th^ paragraph, pp.21).

#3 The authors allude to cultural considerations in Taiwan that may impact the decision to enroll in hospice care. Do the authors think these are significant enough to make the groups different in their decision-making processes? In other words, do individuals who choose hospice care to have different approaches to their health care that bring about the cost differences? What do the authors think we should do with this data?
**Remedy:** Thanks for your comment. The cultural considerations, including the religions, traditional philosophies and values, and family-related barriers in telling the truth strongly influence Taiwanese attitudes toward death, and make many Taiwanese reluctant to talk or think about death and then impact hospice decision making. It is truly the limitation of the study that the data of cultural considerations could not be got from the national health insurance database. Future, the questionnaires which cultural consideration in EOL patient link to the database of the national health insurance database for control the influence factor of cultural consideration. (Please refer to “Discussion,” the 9^th^ paragraph, pp.22).

**Response to Reviewer 2**

#1 The introduction needs additional work by an English-language editor for flow and style. The last two sentences of the first paragraph are editorial comments and appear out-of-place in a manuscript like this.

**Remedy:** Thanks for your comment.

We have had the main text English-edited for better fluency. Besides, we have deleted the last two sentences of the first paragraph according to your valuable comment. (Please refer to “Introduction,” the 1^st^ paragraph, pp.5).

#2 Additional context about the status of and recent changes in hospice care provision in Taiwan should be in the introduction. I would recommend moving the discussion of the development of the hospice shared-care program from the discussion to the intro. An explicit description of the hospice shared-care population in the background would also provide needed context for analyzing the results. Overall, I think the background should include more of an "environmental scan" of end-of-life care in Taiwan: where does it occur, what are the barriers, and why is it important to analyze the cost factors addressed by this paper.

**Remedy:** Thanks for your kind advice. We have moved the content regarding the development of the hospice shared-care program from the discussion section to the introduction section. (Please refer to “ Introduction,” the 2^nd^ paragraph, pp.5-6).

#3 Is it possible to separate out your results and images based on the various types of services that have been lumped together into "hospice" within the study?

**Remedy:** Thanks for your valuable suggestion. We have added the information for various types of hospice care from the total amount. (Please refer to “Results - Medical Expenses for Terminal Patients in Traditional Care or Hospice Care,” the 1^st^ paragraph, pp.16).

#4 It would be particularly powerful in figure 5 to see shared-care hospice as a separate trend line. If this analysis isn't available, I strongly encourage a follow-up study for future publication.

Remedy: Thank you very much for your great advice. The date of hospice-shared care is unavailable from NHIRD, and the hospice-shared care was the service of palliative consultation in the current study. Thus, the medical health expense of hospice-shared care was calculated by the proportion of hospitalization day, and the cumulative cost saving of hospice-shared care cannot separate from all hospice patients. (Please refer to “Discussion,” the 9^th^ paragraph, pp.22-23).

#5 In referring to "inpatient palliative care," for instance, on page 8 when you describe the "hospice group," does this refer to the provision of inpatient expert palliative care team consultation on all those with serious illness (see the WHO definition of palliative care)? Or is this being used to describe those who received "hospice" end-of-life services in the hospital setting? If it is the latter, it needs to describe it that way rather than using the "inpatient palliative care" terminology so that it is more clear to an international audience what is being described. I would also point out that it appears to be described as "inpatient hospice care" in Table 1.

**Remedy:** Thanks for your advice. The “inpatient palliative care” in the current study was used to describe those who received "hospice" end-of-life services in the hospital setting. (Please refer to “Materials and Methods - Research Design and Sample Selection,” the 1^st^ paragraph, pp.7 and“Materials and Methods - Sample Matching,” the 1^st^ paragraph, pp.8).

#6 In section 2, you discuss a 10-day interval; a quick review of the accompanying citation (24570105) doesn't provide a 10-day interval, can there be more explanation of how this number was selected.

**Remedy:** Thanks for your comment. The survival days of terminal cancer patients were a high proportion between 7-10 days (39-51%) after receiving hospice-shared care. We have added this information to the text. (Please refer to “ Materials and Methods - Sample Matching,” the 1^st^ paragraph, pp.8).

#7 Figure 2 needs better grayscale formatting (more like figures 4 or 5).

Remedy: Thanks for your advice. We have revised the figure 2 for better grayscale formatting. (Please refer to “Figure 2”).
